# Solitary Mastocytoma of the Vulva

**DOI:** 10.1155/2014/412656

**Published:** 2014-03-30

**Authors:** Shasi Velusamy, Jayasree Karuthedath Areeppurath Mana, Chalissery Francis Mathew

**Affiliations:** Department of Pathology, Government Medical College, Thrissur, Kerala 680596, India

## Abstract

Solitary mastocytoma of the skin is a rare tumor. Its occurrence in the vulva is extremely rare with only few case reports in the literature. We report a solitary mastocytoma of the vulva in a 10-year-old girl. Her systemic examination was unremarkable. The clinical and histopathological features were consistent with the diagnosis of solitary mastocytoma of the vulva. The follow-up after surgical excision was uneventful. The purpose of this communication is (i) to report a case of solitary mastocytoma occurring in an unusual site, the vulva and to show that (ii) in this case age at presentation is 10 years with infancy as common age of presentation of solitary mastocytoma, and to show that (iii) in appropriate setting this should be included in the differential diagnosis of vulval swelling.

## 1. Introduction

Mastocytoma is a localized collection of benign mast cells in the dermis. Mastocytoma usually appears as a solitary lesion. The common locations are trunk, neck, and arms [1]. The lesion appears at birth or in the first few months of life [2]. This case is described here because of age at presentation and its occurrence in unusual site, the vulva.

## 2. Case History

**Figure 1:**
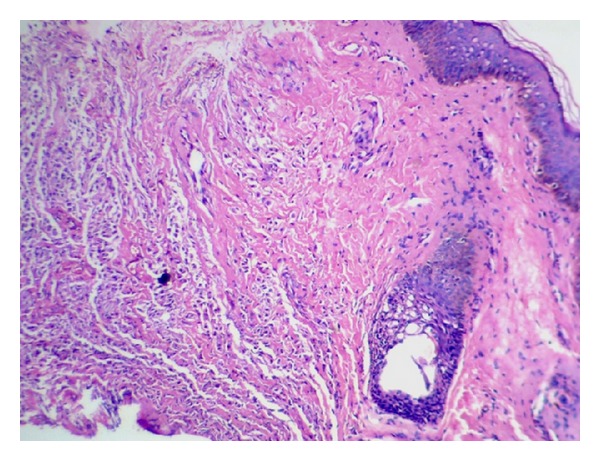
H&E stain. Skin with infiltrate of mast cells in the deep dermis.

**Figure 2:**
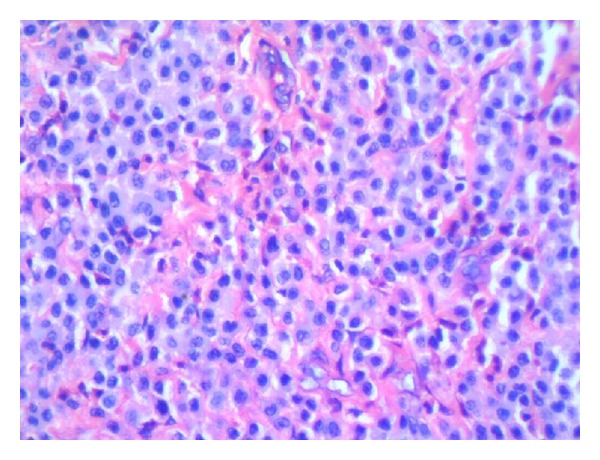
H&E stain. Mast cells with pale blue cytoplasm and round to oval nuclei.

**Figure 3:**
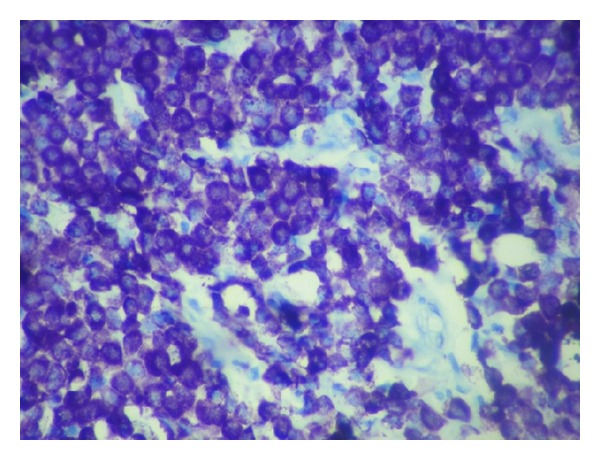
Toluidine blue stain. Mast cells with purple metachromatic granules in the cytoplasm.

**Figure 4:**
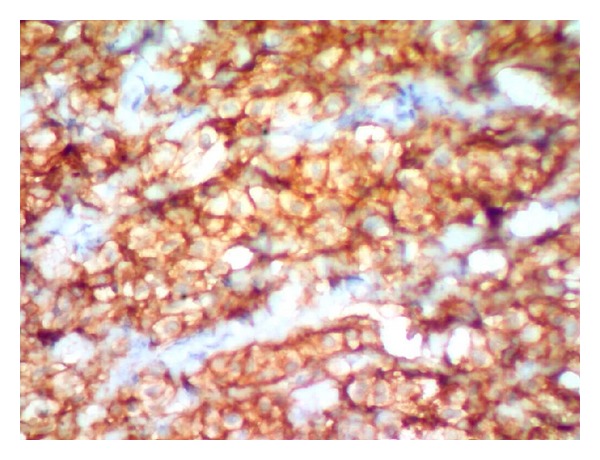
CD 117 IHC. Membrane staining of mast cells.

A 10-year-old girl was brought to the hospital with complaints of recurrent swelling in the labium majus. She complains of itching which usually lasts for a day. The swelling has recurred four times. Except for the labial lesion, physical examination was unremarkable; no hepatosplenomegaly, lymphadenopathy, or skin rash was documented. Excision of the mass was performed. Gross was a single skin covered soft tissue measuring 2.3 × 1.3 × 0.4 cm. Cut section was grey white. On microscopic examination, the mass was composed of a dense monomorphic infiltrate of tumor cells in the dermis and subcutaneous tissue (Figure 1). Cells were medium sized with regular nuclear contours and abundant pale blue cytoplasm (Figure 2). Mitotic activity was absent. Mature eosinophils were scattered in the lesion. A differential diagnosis of Langerhan's cell histiocytosis and mastocytoma was considered. Cytoplasmic granules of the neoplastic cells were strongly metachromatic on Toluidine blue stain (Figure 3) and purple on Giemsa stain. The infiltrating cells were CD117 positive on immunohistochemistry (Figure 4). A diagnosis of solitary mastocytoma of vulva was made.

## 3. Discussion

Swelling in the vulva in this age is uncommon and presents a diagnostic challenge. The swelling was subjected to an excision biopsy considering the possibility of malignancy. Solitary mastocytoma of the skin represents a relatively rare dermal tumour [3]. There is predilection for the trunk, and the other rare sites are eyelids [3], palm [4, 5], orbit [6], and vulva [7, 8]. Vulva is a rare site for this tumour. Solitary mastocytoma is one of three variants of cutaneous mastocytosis. The three variants of cutaneous mastocytosis are UP/maculopapular cutaneous mastocytosis, solitary mastocytoma, and diffuse cutaneous mastocytosis [2]. Solitary lesions may involute spontaneously without surgical excision [9, 10]. In this case also there is history of recurrent swelling four times in the same site which involuted spontaneously without treatment. Solitary mastocytoma presents as macules, plaques, or nodules and is formed by dermal collection of mast cells. Cellular atypia is not detected. This allows separation of mastocytoma from an extremely rare mast cell sarcoma of the skin. Giemsa or Toluidine blue stain is employed to detect the metachromatic mast cell granules and chloroacetate esterase (CAE) is also helpful. Most specific methods are immunohistochemical staining for tryptase/chymase and CD 117 and for neoplastic mast cells CD2 and CD25 [2]. When systemic symptoms are present, they most commonly involve flushing [10]. The course of the solitary mastocytoma is benign. The precise cause of this disease is unknown, but tumor resection is effective and prognosis is favorable.

## 4. Conclusion

Mastocytoma of the vulva is very rare. The unique aspects of this case include its site, age of presentation, and the history of recurrent swelling in the same site before its current presentation. So in appropriate setting this should be included in the differential diagnosis of vulval swelling. In this case, the postoperative period was uneventful and there is no recurrence on follow up.

## Key Messages

A mastocytoma localized to the vulva is an extremely rare condition; only a few known cases of this condition have been reported so far. Awareness and inclusion in the differential diagnosis are important.
